# Tumor Necrosis Factor-α G-308A Polymorphism and Sporadic IgA Nephropathy: A Meta-Analysis Using a Genetic Model-Free Approach

**DOI:** 10.3390/genes14071488

**Published:** 2023-07-21

**Authors:** Maria Tziastoudi, Ioanna Chronopoulou, Georgios Pissas, Christos Cholevas, Theodoros Eleftheriadis, Ioannis Stefanidis

**Affiliations:** 1 Department of Nephrology, Faculty of Medicine, School of Health Sciences, University of Thessaly, 41500 Larissa, Greece; ichron@yahoo.gr (I.C.); gpissas@msn.com (G.P.); teleftheriadis@yahoo.com (T.E.); stefanid@med.uth.gr (I.S.); 2 First Department of Ophthalmology, Faculty of Health Sciences, Aristotle University of Thessaloniki School of Medicine, AHEPA Hospital, 54636 Thessaloniki, Greece; ccholevas@auth.gr

**Keywords:** immunoglobulin A nephropathy (IgAN), tumor necrosis factor-α, TNF-α, polymorphism, meta-analysis

## Abstract

Tumor necrosis factor-α (TNF-α) is a potent pro-inflammatory cytokine, involved in the pathogenesis and progression of immunoglobulin A nephropathy (IgAN). A bi-allelic polymorphism in the promoter region, at position -308 (G/A) of the *TNF*-*α* gene (rs1800629) is associated with an increased TNF-a production. However, several previous association studies of *TNF*-*α* G-308A polymorphism and IgAN rendered contradictory findings. The objective of the present study is to shed light on these inconclusive results and clarify the role of TNF-α and any possible contribution of this factor in the development and progression of sporadic IgAN. Therefore, a meta-analysis of all available genetic association studies relating the *TNF-α* G-308A polymorphism to the risk for development and/or progression of IgAN was conducted. Seven studies were included in the meta-analysis. Three of them included populations of European descent (Caucasians) and four involved Asians. The generalized odds ratio (OR_G_) was used to estimate the risk for the development and/or progression of the disease. Overall, the meta-analysis did not detect any significant association between the G-308A variant and both the risk of developing IgAN and the risk for progression of IgAN. In conclusion, these results suggest that *TNF-α* does not constitute a key component in the genetic architecture of sporadic IgAN. However, further evidence deciphering the influence of *TNF-α* on IgAN is still needed.

## 1. Introduction

Immunoglobulin A nephropathy (IgAN) is the most common primary glomerulonephritis for which only supportive care is available up to date [1]. It is characterized by prominent glomerular, primarily mesangial, deposits of polymeric subclass 1 IgA (IgA1), accompanied by mesangial cell proliferation, extracellular matrix accumulation and glomerular infiltration, predominantly consisting of monocytes and macrophages [2]. Many clinical and histological markers strive to predict the course of the disease. More specifically, clinical biomarkers include the presence of hypertension, proteinuria, high body mass index (BMI) and smoking, whereas histological markers refer to the Oxford MEST-C score which uses five indicators (mesangial and endocapillary hypercellularity, segmental sclerosis, interstitial fibrosis/tubular atrophy, and the presence of crescents) [3,4,5].

However, new biomarkers specific to the disease are needed for a more reliable estimation of risk. Genetic studies, such as genome-wide association studies (GWAS), have shed light on several variants, many of which are harbored in immunologic and inflammatory pathways [6,7,8,9,10,11,12], among which many are related to mucosal immune response, innate and adaptive immunity, as well as the complement activation [13]. More specifically, the study of Li et al. identified three novel loci, *FCRL3*, *DUSP22*.*IRF4*, and *PADI4*, three *HLA* polymorphisms and two variants harbored in the MHC region as susceptibility genes of IgAN [6]. Another study in Han Chinese identified associations at 17p13 (rs3803800), 8p23 (*TNFSF13* and *DEFA*), 22q12 and multiple associations in the major histocompatibility complex (MHC) region [7]. *ST6GAL1*, *ACCS*, *ODF1*-*KLF10*, *ITGAX*-*ITGAM* and *DEFA* were highlighted as risk loci of IgAN [8]. One more GWAS investigated the common susceptibility loci of IgAN and systemic lupus erythematosus (SLE) and identified 14 loci common between IgAN and SLE [9]. *ITGAM-ITGAX, VAV3, CARD9, HLA-DQB1* and *DEFA* were also revealed as significant risk loci of IgAN proposing a potential influence of interactions between intestinal pathogens and the host on the genetic makeup of IgAN [10]. Rs2296136 in *ANKRD16* was also significantly associated with IgAN in Koreans [12].

A crucial role in this process has been shown for various inflammatory cytokines including tumor necrosis factor α (TNF-α), which is a potent mediator with a central role in the inflammatory cascade [14]. There is recent experimental evidence that in IgAN mesangial cells derived TNF-α might be involved in the pathogenesis of tubular damage and interstitial fibrosis [15,16], both closely related to disease progression [17]. It is therefore conceivable to hypothesize a role of TNF-α in the pathogenesis and progression of IgA nephropathy. More specifically, recent findings suggest that the release of TNF-α from the mesangium following IgA deposition triggers the activation of renal tubular cells. This communication between the glomerulus and tubules may have a significant impact on the development of tubulointerstitial damage in IgAN. Based on in vitro results, it is indicated that podocytes may contribute to the progression of interstitial damage in IgAN by enhancing the activation of tubular epithelial cells through increased synthesis of TNF-a following inflammatory changes in human mesangial cells (HMC) [15,16].

The tumor necrosis factor-α gene (*TNF-α*; OMIM 191160) is located on the short arm of chromosome 6 (6p21.33) [18,19]. A bi-allelic polymorphism has been long characterized in the promoter region of *TNF-α*, namely a G→A transition at position -308 [20]. Carriage of the infrequent allele A, in comparison to the allele G, is associated with increased gene transcription [20,21] and elevated TNF-α production by lymphocytes in vitro [22]. The association of the *TNF-α* G-308A polymorphism and IgAN has been investigated in several genetic studies [23,24,25,26,27,28,29], that rendered contradictory findings. Concretely, some of the studies indicated an association of the polymorphism with IgAN [23,24,25,28] and others urged that there is no association [26,27,29]. Among studies with significant results, one study did not detect an association with IgAN per se but only with gross hematuria [24], one study reported an association which was not remained significant after the application of Bonferroni correction [28], one study revealed a protective role of -308A allele in IgAN [25], whereas another study indicated -308A allele as a potent risk factor for the progression of the disease [23].

Although the conflicting significant results about the contribution of G-308A polymorphism in the course of IgAN, in an effort to reconcile these findings and provide a resolution, a meta-analysis of case–control studies evaluating the *TNF-α* G-308A polymorphism to the risk for development and progression of sporadic IgAN was conducted.

## 2. Material and Methods

### 2.1. Selection of Studies

In order to clarify the contribution of *TNF-α* in sporadic IgAN susceptibility and progression, we meta-analyzed all case–control studies regarding the G-308A polymorphism in the *TNF-α* gene in the context of sporadic IgAN published before June 2023. The studies were retrieved after an extensive search of the PubMed, Scopus, ScienceDirect, Web of Science and Wiley Online Library databases using the search terms [“tumor necrosis factor” AND “IgA nephropathy” AND polymorphism] (accessed on 24 June 2023).

The retrieved publications were thoroughly reviewed to evaluate their eligibility. All references of the eligible studies were scrutinized to identify articles not indexed in the aforementioned databases. Abstracts, case reports, editorials, review articles, in vitro studies, as well as family-based studies were excluded. It is important to note that the search included only articles in English. Two investigators (M.T. and I.S.) evaluated the eligibility of the articles, and any disagreements were resolved through consensus.

In the association studies, that were eligible for meta-analysis concerning progression of IgA nephropathy, both cases and controls were patients with sporadic IgA nephropathy. Patients with a progressive IgAN were considered as cases (progressors) and those with stable nephropathy as controls (non-progressors). Eligibility was not dependent on a specific prefixed definition of IgAN progression. The definition of progression used in each study was accepted and is presented in Table 1. Subjects with other forms of IgAN, such as Henoch–Schönlein purpura, which is the systemic form of IgAN, and subjects with secondary IgAN were excluded.

### 2.2. Data Extraction

The following details were extracted from each study: the primary author, publication year, racial background of the study participants, selection criteria, demographic information, and complete genotype counts. Allele frequencies were calculated from genotype counts.

### 2.3. Data Synthesis and Analysis

To examine the connection between genotype distribution and the risk of developing sporadic IgAN or the risk of disease progression, the generalized linear odds ratio (ORG) was used [30,31]. Meta-analysis was conducted when at least two studies were available. The pooled odds ratio (OR) was estimated using random effects models (DerSimonian and Laird) [32]. All associations were reported as odds ratios (OR) with their corresponding 95% confidence intervals (Cis). Heterogeneity between studies was assessed using the Q-statistic [33] and the degree of heterogeneity was quantified using the I^2^ metric [34]. Software implementing the generalized odds ratio methodology was used (ORGGASMA: https://biomath.med.uth.gr/default.aspx?lang=el&id=232164AC-9C6B-4A27-A595-2A22C35B6260&rid=576AB0F4-10AE-4BEA-8D97-C52B8B6BD4DA) (accessed on 1 May 2023) [30].

The distribution of the genotypes in the control group was tested for deviation from the Hardy–Weinberg equilibrium (HWE) using Fisher’s exact test. We also tested for small-study effects with the Egger test [35].

The meta-analysis comprised three components: the main analysis, encompassing all accessible data; subgroup analysis, considering each racial population separately; and sensitivity analyses, investigating the impact of excluding studies that were not confronted with the Hardy–Weinberg equilibrium (HWE).

All analyses were performed using the Comprehensive Meta-analysis software package (CMA version 4; http://www.meta-analysis.com; 2005) (accessed on 1 May 2023) and StatsDirect software (StatsDirect Ltd., Wirral, UK, StatsDirect statistical software. http://www.statsdirect.com. England: StatsDirect Ltd. 2008) (accessed on 1 May 2023).

## 3. Results

### 3.1. Study Characteristics

**Figure 1:**
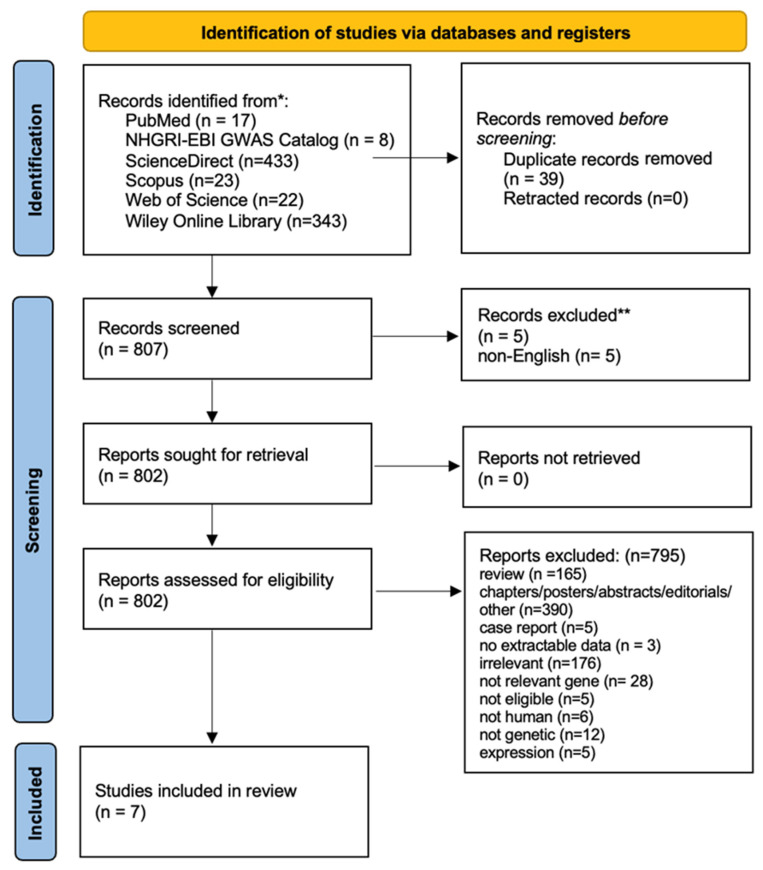
PRISMA 2020 flow diagram of retrieved articles with specifications of reasons for exclusion.

The literature review identified 846 titles in all databases. After the removal of the duplicates and a thorough review of the articles, seven studies were included in the meta-analysis. Figure 1 presents a flow chart of the retrieved articles, as well as the excluded articles with the reasons for exclusion. More specifically, seven studies investigated the association between *TNF-a* gene G-308A polymorphism (rs1800629) and sporadic IgAN susceptibility, whereas five studies investigated the association of the polymorphism to the risk of progressive IgAN. The study characteristics are described in Table 1.

In all studies investigating the association of the polymorphism with sporadic IgAN the patients with IgAN were well defined following identical inclusion criteria. The existence of IgAN was documented histologically on the basis of a kidney biopsy logically accompanied by immune-fluorescence microscopy. Studies that included only transplanted patients were excluded. The controls were healthy individuals from the same region or healthy blood donors (Table 1). Subjects with other forms of IgAN, such as Henoch–Schönlein purpura and secondary IgAN were excluded.

Criteria applied to define progression were not identical in five studies investigating the association of the polymorphism with the progression of IgA nephropathy. However, per protocol, all were eligible for the meta-analysis (Table 1). For reasons of clarity, the definition of IgAN progression or not is presented in Table 1 for each study included in the meta-analysis.

### 3.2. Summary Statistics

In total, the studies examining the association of the *TNF-α* G-308A polymorphism with IgAN included 1486 cases (with IgA nephropathy), and 1715 healthy controls, whereas the studies that examined only the association of the *TNF-α* G-308A polymorphism with progression of IgAN included overall 196 cases (progressors), and 526 controls (non-progressors). In one study [25], the distribution of the genotypes in the control group was not confronted with HWE (*p* < 0.05), indicating population stratification or genotyping errors. Therefore, a sensitivity analysis was carried out for this study. Table 2 shows the distribution of the *TNF-α* genotypes for patients with sporadic IgAN and healthy controls, whereas Table 3 shows the distribution of the *TNF-α* genotypes for patients with progressive IgAN and patients with non-progressive IgAN.

### 3.3. Meta-Analyses Results

#### Risk of Sporadic IgAN

The main analysis, in which seven studies were included, did not reveal statistically significant results [OR_G_ = 0.80 (95% CI 0.56–1.14)]. Similarly, no significant association was detected in sensitivity analysis in which only studies confronted with HWE were included [ORG = 0.94 (95% CI 0.70–1.26)] (Table 4).

In subgroup analyses regarding the Caucasian and Asian populations separately, there was large heterogeneity (I^2^ = 71.92% and 61.83%, respectively) between the studies with a polled ORG of 0.74 (95% CI 0.44–1.26) and 0.85 (95% CI 0.50–1.43), respectively (Table 4).

### 3.4. Risk for Progression of Sporadic IgAN

The main analysis included five studies and did not detect any significant association between G-308A polymorphism and the risk for progression of sporadic IgAN [OR_G_ = 1.13 (95% CI 0.74–1.73)] (Table 4). Similarly, the sensitivity analysis did not also show significant results [OR_G_ = 1.19 (95% CI 0.74–1.92)] (Table 4). Both main and sensitivity analyses were characterized by no heterogeneity (I^2^ = 7.13% and 14.09%, respectively).

**Figure 2:**
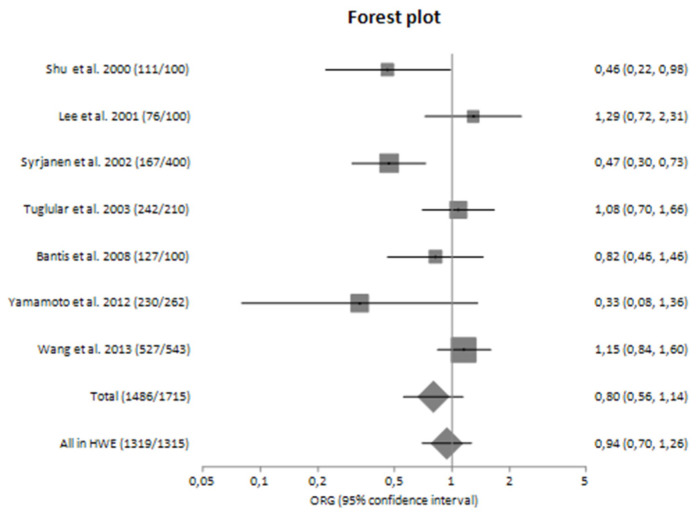
Forest plot of *TNF-α* G-308A polymorphism and the risk of sporadic IgA nephropathy in main analysis [23,24,25,26,27,28,29].

**Figure 3:**
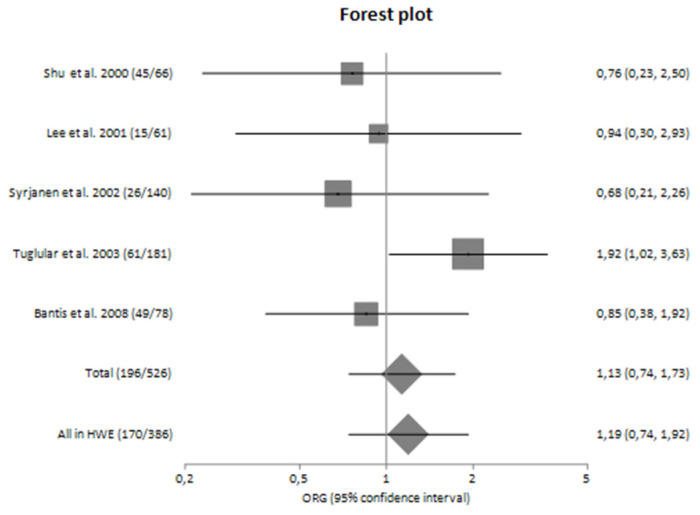
Forest plot of *TNF-α* G-308A polymorphism and the risk for progression of IgA nephropathy in main analysis [23,24,25,26,27,28,29].

In subgroup analyses regarding the Caucasian and Asian populations, no significant association was revealed with a pooled ORG of OR_G_ = 1.16 (95% CI 0.61–2.23) and OR_G_ = 0.85 (95% CI 0.37–1.93), respectively (Table 4). Figure 2 and Figure 3 are forest plot representations of *TNF-a* G-308A polymorphism and the risk of IgAN and the risk for progression of sporadic IgAN, respectively.

**Figure 4:**
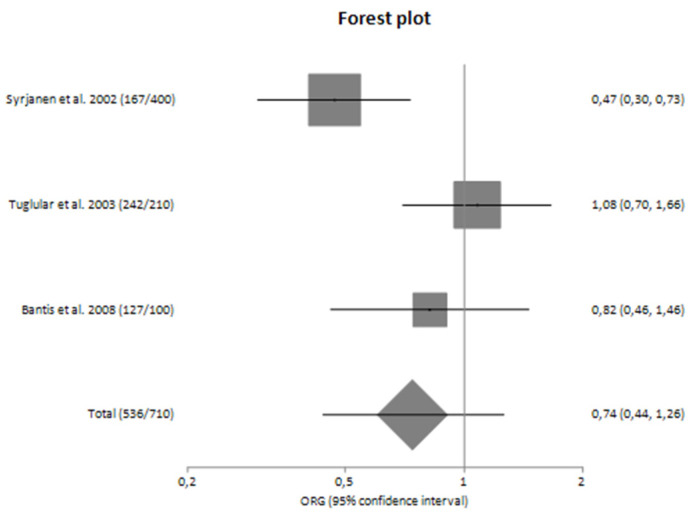
Forest plot of *TNF-α* G-308A polymorphism and the risk of sporadic IgA nephropathy in Caucasians [25,26,27].

**Figure 5:**
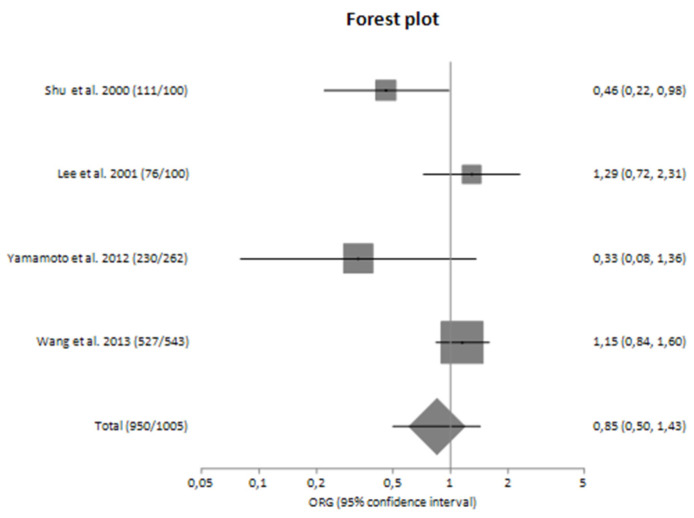
Forest plot of *TNF-α* G-308A polymorphism and the risk of sporadic IgA nephropathy in Asians [23,24,28,29].

**Figure 6:**
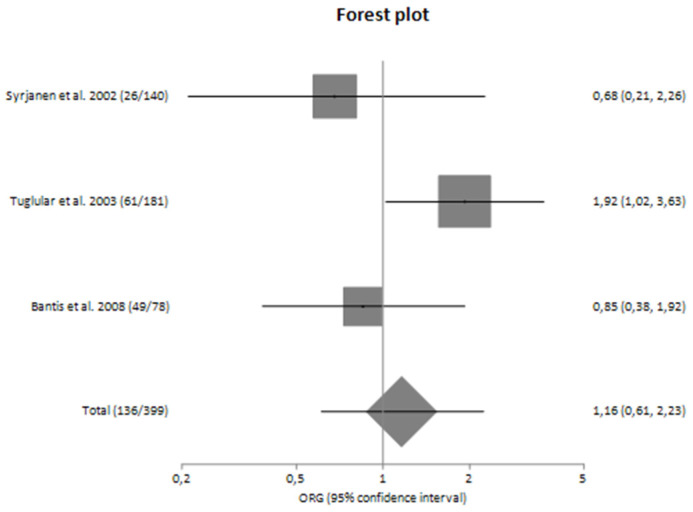
Forest plot of *TNF-α* G-308A polymorphism and the risk for progression of sporadic IgA nephropathy in Caucasians [25,26,27].

**Figure 7:**
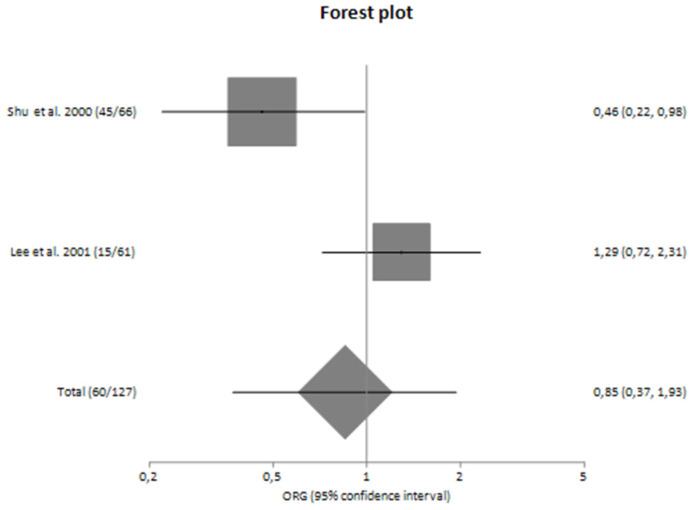
Forest plot of *TNF-α* G-308A polymorphism and the risk for progression of sporadic IgA nephropathy in Asians [23,24].

Figure 4, Figure 5, Figure 6 and Figure 7 are also forest plot representations of *TNF-a* G-308A polymorphism and the risk of IgAN and the risk for progression of sporadic IgAN, respectively in subgroup analyses.

## 4. Discussion

IgAN is the prevailing form of glomerulonephritis, characterized by inflammation affecting both the glomeruli and blood vessels within the kidneys. Despite extensive efforts, its pathogenetic mechanism is not absolutely clear to date. On the basis of the GWAS results, which highlighted the contribution of several variants harbored in immunologic and inflammatory pathways, we conducted a systematic review and meta-analysis to address the potential implication of *TNF-α* G-308A polymorphism in both the susceptibility and progression of sporadic IgAN. More specifically, the present meta-analysis included the genetic association studies that investigated the contribution of G-308A polymorphism in the risk of developing sporadic IgAN and in the risk for progression of sporadic IgAN.

Experimental data revealed that TNF-α is a pleiotropic cytokine with a variety of proinflammatory properties, which is produced by both infiltrating cells, mainly monocytes and macrophages, and mesangial cells [36]. TNF-α serum levels were elevated in patients with IgAN and were also correlated with histological activity [37]. Another study revealed an increased release of TNF-α from the white blood cells of patients with IgAN [38].

On the basis of the available data about *TNF-α*, G-308A polymorphism of this gene has also been studied in several genetic association studies with inconclusive results. Shu et al. (2000) found that G-308A polymorphism was not correlated with disease progression and long-term outcome and did not also correlate with disease activity, except for gross hematuria in Asians, suggesting that *TNF-α* may not play an important role in disease progression [24]. In contrast, Lee et al. (2001) found a statistically significant association between G-308A polymorphism and the progression of IgAN [23]. However, Syrjänen et al. concluded that the carriage of the -308A allele is protective against IgAN, but it did not affect the prognosis of the disease [25]. It is noteworthy to mention the synergistic effect of *IL1b2*, *IL1RN* and *TNF-α* because it was found that the carriage of both *IL1b2* and IL1RN*2 together with the non-carriage of *TNF-α* -308A allele increased the risk of IgAN fivefold [25]. The study of Syrjänen et al. is important because it was conducted in a large sample of a Finnish population, a genetically homogenous population, with a long follow-up period and a low number of patients lost during the follow-up period [25]. Another strength of this study is its policy for renal biopsy even in cases of minor urinary abnormalities [25]. Another study revealed a significant association of G-308A polymorphism with susceptibility but not with the progression of the disease [26]. In addition, according to Bantis et al. (2008), G-308A polymorphism does not constitute a risk factor or marker of progression in Caucasians with IgAN [27]. Yamamoto et al. (2012) also did not reveal the G-308A polymorphism as a potential genetic contributor to IgAN [29]. Finally, in the study of Wang et al. (2013), the G-308A polymorphism exhibited a suggestive association with IgAN [28] which was not significant after the Bonferroni correction was applied. In our meta-analysis, *TNF-α* G-308A polymorphism was not associated with either the susceptibility or progression of sporadic IgAN, either in the main analysis or in sensitivity and subgroup analyses.

These conflicting results are difficult to interpret. The discrepancy in results could be related to racial differences. More specifically, it is known that the prevalence of IgAN is very high in East Asians, intermediate in European Caucasians and low in Africans [39]. It is also noteworthy to mention that the local policies regarding indications for renal biopsy vary among different countries and this fact could affect the proportion of reliable and early diagnosis of the disease. So, different policies could constitute another source of heterogeneity. It would also be very interesting to perform subgroup analyses regarding gender because according to a recent study conducted in Estonia, it has been observed that the advancement of renal disease is more rapid in males compared to females. Additionally, there is a correlation between higher Oxford MEST scores and the progression of the disease in male patients [40,41]. However, this is not feasible because the included studies do not present genotype counts for males and females separately. In addition, the interpretation of the results can be further complicated due to the dual function of TNF-α, as a pro-inflammatory factor in the initial infection and as an anti-inflammatory or immunoregulatory factor in the later phases of the response [25]. Therefore, the increased expression of TNF-α could also reflect the activation of protective mechanisms in the context of severe glomerular injury in an effort to suppress the inflammation. Further heterogeneity is also introduced by differences in patient material, such as the different proportions of patients in each study who have reached end-stage renal disease (ESRD), as well as the different characteristics of each study such as the period of follow-up and the number of patients lost during the follow-up period.

Unfortunately, there is no specific treatment for IgAN to date. Patients with IgAN receive treatment that applies to virtually any glomerular disease [3]. More specifically, the current treatment of IgAN includes supportive care, immunosuppression therapy, as well as alternative therapies to conventional immunosuppression [13]. Regarding supportive care, it comprises medications for lowering blood pressure and reducing proteinuria, along with comprehensive education on lifestyle modifications and the importance of avoiding combinations of medications that can harm the kidneys [13]. Therefore, angiotensin-converting enzyme (ACE) inhibitors and angiotensin receptor blockers (ARBs) constitute the first-line treatment for IgAN. Currently, there is no evidence suggesting that combining ACE inhibitors and ARBs should be avoided in IgA nephropathy (IgAN) [13]. The armamentarium of the single renin–angiotensin system (RAS) blockade is further expanded with the use of sparsentan, a dual endothelin angiotensin receptor antagonist (DEARA) whose reno-protective effects were evaluated in PROTECT trial versus irbesartan [42]. Furthermore, sodium–glucose cotransporter-2 inhibitors (SGLT2i) constitute a novel therapeutic approach with promising results, although the precise mechanism of action has not been deciphered yet [43,44].

The second category of therapeutic interventions of IgAN involves immunosuppressive agents. According to the KDIGO guidelines, IgAN patients who continue to be at a high risk of disease progression even after receiving optimized supportive care for at least 90 days should contemplate undergoing a six-month regimen of systemic corticosteroid therapy [13]. Nevertheless, the effectiveness and safety of corticosteroids remain a subject of debate and uncertainty [45]. Except for corticosteroids, mycophenolate mofetil (MMF) has been evaluated in several studies in populations of different racial descent, leading to conflicting results. More specifically, in Caucasians it showed no benefit, whereas in Chinese patients with IgAN, it reduced proteinuria and retarded the decline of eGFR [46,47,48], although several cases of severe pneumonia were reported in a cohort of Chinese patients [49]. Similarly, cyclophosphamide produced inconclusive data and, as a result, the KDIGO guideline does not endorse its utilization in the majority of patients with IgAN [3]. It is not also recommended the use of azathioprine in IgAN [3].

It is noteworthy to mention that many ongoing trials are performed in an effort to develop new therapeutic agents for the treatment of IgAN and new pathogenetic pathways are revealed [47,50,51,52]. These new approaches include agents that target the regulation of pathogenic IgA1 and CIC production, like inhibition of TLRs/BAFF/APRIL signaling (hydroxychloroquine, blisibimod, VIS649, BION-1301, atacicept and telitacicept) and the depletion of Gd-IgA1-producing plasma cells, the clearance of IgA deposits (fostamatinib) and the modulation of mucosal immunity, such as the modulation of NALT and the modulation of GALT and gut microbiota [13,50]. Last but not least, a case report reported the beneficial effects of aliskiren, the first direct inhibitor of renin activity [53].

In this study, we used the generalized odds ratio (OR_G_) to estimate the magnitude of the association. This metric overcomes the problem of multiple comparisons of the different genetic models (dominant, recessive, additive, co-dominant, and allele contrast) by utilizing the full genotypic counts and prevents confusion in cases when more than one genetic model is significant. Therefore, the interpretation of the results is straightforward and more robust. In addition, the choice of a specific genetic model a priori is not required. This metric has been used in various studies of other diseases, like diabetic nephropathy [54,55,56,57,58].

## 5. Conclusions

In conclusion, the current meta-analysis reviews the latest genetic epidemiology findings regarding the contribution of G-308A polymorphism in the risk of developing IgAN and the progression of IgAN. Since this meta-analysis did not detect any significant association of G-308A polymorphism with risk for the development or progression of IgAN, we conclude that this genetic component does not constitute a key genetic component in the pathogenesis of sporadic IgAN at least alone. However, the results should be interpreted with caution because the number of studies in the meta-analysis is relatively small.

## Figures and Tables

**Table 1:** Characteristics of the case–control studies considered in the meta-analysis.

			IgA Nephropathy				HWE (*p*-Value)	Progression	of IgA Nephropathy		
First Author, Year	Country	Racial Descent	Selection Criteria and Demographic Data of Cases	Selection Criteria and Demographic Data of Healthy Controls	Cases (n)	Controls (n)		Selection Criteria and Demographic Data of Cases (Progressors)	Selection Criteria and Demographic Data of Controls (Non-Progressors)	Cases (n)	Controls (n)
Shu, 2000 [24]	China	Asians	Biopsy-proven IgA nephropathy (57 males, 54 females, mean age 30.3 years). Cases with Henoch–Schoenlein purpura not mentioned.	Healthy controls not matched to cases for age and gender; further demographic data not mentioned.	111	100	0.5	Increase of serum creatinine or more than 50% increase of daily proteinuria or appearance of hypertension.	Patients with stable renal disease or those in remission.	45	66
Lee, 2001 [23]	Korea	Asians	Biopsy-proven IgA nephropathy (38 males, 38 females, mean age 30.4 years), no evidence of primary causes. Cases with Henoch–Schoenlein purpura not mentioned.	Healthy controls (49 males, 51 females, mean age 48.2 years) from the same region; not matched to cases for age and gender.	76	100	0.9	Patients with doubling of serum creatinine in comparison to the initial evaluation or start of hemodialysis treatment for end-stage renal disease at follow-up.	Patients without doubling of serum creatinine and no start of hemodialysis treatment for end-stage renal disease at follow-up.	15	61
Syrjanen, 2002 [25]	Finland	Caucasians	Biopsy-proven IgA nephropathy (102 males, 65 females), no evidence of primary causes. Nine cases with Henoch–Schoenlein purpura.	Healthy blood donors (100 males, 100 females) from local center; not matched to cases for age and gender.	167	400	0.04	Presence of chronic renal failure (serum creatinine ≥125 μmol/L in males and ≥105 μmol/L in females) initially or rise of serum creatinine over 20% at follow-up.	Absence of chronic renal failure (serum creatinine ≥125 μmol/L in males and ≥105 μmol/L in females) initially or at follow-up.	26	140
Tuglular, 2003 [26]	France	Caucasians	Biopsy-proven IgA nephropathy (169 males, 73 females), no evidence of primary causes. No cases with Henoch–Schoenlein purpura.	Healthy controls (133 males, 77 females) from the same region; not matched to cases for age and gender.	242	210	0.22	Presence of chronic renal failure according to K/DOQI Clinical Practice Guidelines (GFR < 60/min × 1.73 m^2^ by Cockroft–Gault equation) initially or at follow-up.	Absence of chronic renal failure according to K/DOQI Clinical Practice Guidelines (GFR < 60/min × 1.73 m^2^ by Cockroft–Gault equation) initially or at follow-up.	61	181
Bantis, 2008 [27]	Germany	Caucasians	Biopsy-proven IgA nephropathy (96 males, 31 females, mean age 37.7 years). Cases with Henoch–Schoenlein purpura not mentioned.	Volunteers without kidney diseases or arterial hypertension matched for age; further demographic data not mentioned.	127	100	0.76	Yearly change in the reciprocal of serum creatinine levels lower than −0.1 mg^−1^dL (38 males, 11 females, mean age 36.6 years).	Yearly change in the reciprocal of serum creatinine levels higher than −0.1 mg^−1^dL (58 males, 20 females, mean age 38.4 years).	49	78
Yamamoto, 2012 [29]	Japan	Asians	Biopsy-proven IgA nephropathy patients aged between 25 and 50 years	Healthy hospital employees aged between 25 and 50 years.	230	262	0.80	-	-	-	-
Wang, 2013 [28]	China	Asians	Biopsy-proven primary IgAN with no evidence of systemic diseases such as diabetes, chronic liver disease and systemic lupus erythematosus.	Gender and age matched healthy controls with no history of renal disease or hypertension.	527	543	0.45	-	-	-	-

HWE: Hardy–Weinberg Equilibrium.

**Table 2:** The distribution of the *TNF-α* genotypes for patients with IgA nephropathy (IgAN) and healthy subjects without IgA nephropathy (healthy controls) are shown.

				Risk for Sporadic IgA Nephropathy					
				Distribution of *TNF-α* Genotypes					
First Author	Year	Racial	Cases/ Controls	GG		GA		AA	
		Decent		IgAN	Controls	IgAN	Controls	IgAN	Controls
Shu	2000	Asian	111/100	99	79	11	19	1	2
Lee	2001	Asian	15/61	45	65	26	31	5	4
Syrjanen	2002	Caucasian	26/140	138	275	28	120	1	5
Tuglular	2003	Caucasian	61/181	185	164	54	41	3	5
Bantis	2008	Caucasian	127/100	96	71	26	27	5	2
Yamamoto	2012	Asian	230/262	228	254	2	8	0	0
Wang	2013	Asian	527/543	443	461	82	80	8	2

**Table 3:** The distribution of the *TNF-α* genotypes for patients with progressive IgA nephropathy (cases with progressive IgAN; PR) and patients with non-progressive IgAN (diseased controls; NPR) are shown.

				Risk for Progression of Sporadic IgA Nephropathy					
				Distribution of *TNF-α* Genotypes					
First Author	Year	Racial	Cases/ Controls	GG		GA		AA	
		Decent		Pr	NPr	Pr	NPr	Pr	NPr
Shu	2000	Asian	45/66	41	58	4	4	0	4
Lee	2001	Asian	76/100	10	35	2	24	3	2
Syrjanen	2002	Caucasian	167/400	23	114	3	25	0	1
Tuglular	2003	Caucasian	242/210	41	144	20 *	37 *		
Bantis	2008	Caucasian	49/78	38	58	9	17	2	3

* Data concerned AA + GA.

**Table 4:** The association of the *TNF-α* G-308A polymorphism and the risk of sporadic IgA nephropathy: summary estimates for the odds ratio (OR) of various allele/genotype contrasts, the significance level (*p*-value) of the heterogeneity test (Q-test) and the I^2^ metric in overall analysis, as well as in the subgroup and sensitivity analyses.

GENE	VARIANT	RS	Studies (n)	Cases/Controls (n)	RE OR_G_	95% LL	95% UL	I^2^ (%)	P_Q_	P_E_
Risk for IgAN										
*TNF-α*	-308G > A	rs1800629	7	1486/1715	0.80	0.56	1.14	65.98	0.01	0.40
		All in HWE	6	1319/1315	0.94	0.70	1.26	40.71	0.13	0.65
		Caucasians	3	536/710	0.74	0.44	1.26	71.92	0.03	0.97
		Asians	4	950/1005	0.85	0.50	1.43	61.83	0.05	0.54
**Risk for progression of IgAN**										
*TNF-α*	-308G > A	rs1800629	5	196/526	1.13	0.74	1.73	7.13	0.37	0.40
		All in HWE	4	170/386	1.19	0.74	1.92	14.09	0.32	0.64
		Caucasians	3	136/399	1.16	0.61	2.23	43.80	0.17	0.97
		Asians	2	60/127	0.85	0.37	1.93	0.00	0.81	-

LL: lower limit, UL: upper limit.

## Data Availability

The datasets used and/or analyzed during the current study are available from the corresponding author.
